# HDAC Inhibitor, CG-745, Enhances the Anti-Cancer Effect of Anti-PD-1 Immune Checkpoint Inhibitor by Modulation of the Immune Microenvironment

**DOI:** 10.7150/jca.44622

**Published:** 2020-04-06

**Authors:** Young-Dae Kim, Sang-Min Park, Hae Chan Ha, A Reum Lee, Heeyoung Won, Hyunju Cha, Sangsook Cho, Joong Myung Cho

**Affiliations:** Institute for Drug Discovery, CrystalGenomics, Inc., Korea Bio Park, 700 Daewangpangyo-ro, Bundang-gu, Seongnam-si, Gyeonggi-do 13488, Korea

**Keywords:** CG-745, HDAC, PD-1, TME, Treg, MDSC, Hepatocellular carcinoma, Colorectal cancer

## Abstract

Histone deacetylase inhibitors (HDACis) are well-known epigenetic regulators with therapeutic potential in various diseases. Recent studies have shown that HDACis are involved in immune-mediated anti-cancer effects and may modulate the activity of immunotherapy agents. CG-745, a histone deacetylase inhibitor, has shown anti-cancer effects in pancreatic cancer, colorectal cancer, and non-small cell lung cancer. However, the exact role of CG-745 within the immune system is largely unknown. In this study, we have shown that CG-745 induces microenvironment changes promoting anti-cancer effect of anti-PD-1 antibody in syngeneic mouse models. Specifically, CG-745 induces or extends IL-2 and IFN-γ expression with or without additional stimulation, and increases proliferation of cytotoxic T cells and NK cells, while inhibiting proliferation of regulatory T cells. The analysis of immune cell distribution in the tumor microenvironment and spleen reveals that CG-745 suppresses M2 macrophage polarization and decreases the myeloid-derived suppressor cells. Recent advances in immunotherapy highlight the anti-cancer effects of immune checkpoint inhibitor despite a relatively limited clinical benefit in the subset of patients. Our results indicate that CG-745 enables the synergistic effects of the immune checkpoint inhibitor combination therapy in various cancers by suppressing tumor microenvironment.

## Introduction

While the use of immune checkpoint inhibitors (ICI) has shown significant efficacy in certain types of cancer, especially in melanoma 1, immunotherapies using ICI still face difficulties in controlling malignancy in many types of cancer due to the heterogeneity and individual patient's genetic makeup 2. Activation of T cells results in the induction of immune checkpoint receptor expression (e.g., PD-1, PD-L1, CTLA4, TIM-3, and LAG-3) which occurs at the T cell-antigen-presenting cell interface, reaching a level where it eventually blocks co-stimulation and abrogates an activated T cell's response 3. Based on expression of immune checkpoint receptors, ICI interrupts and removes inhibitory signals of T-cell activation that causes tumor-reactive T cells to overcome regulatory mechanisms and produce an effective anti-cancer response 4, 5. Therefore, ICI will not be able to block its interaction unless the T cell is immunologically inactive. It is also well known that the hostile immunosuppressive tumor microenvironment is a key barrier to the anti-tumor efficacy of ICI 6. In addition, one strategy to overcome this hostile environment is a combination of ICI with immune-modulatory agents 7, 8. For this reason, identification of drugs with immunotherapeutic benefits is being actively studied.

Histone deacetylase inhibitors (HDACis) are also under study as a candidate agent for treatment of certain cancer types 9-11. Histone acetylation modifies chromatin organization and largely affects gene expression regulation 12. Histone acetyltransferases (HATs) add acetyl groups to the lysine residues on target proteins including histones, leading to loosening of chromatin conformation and promoting transcription. On the other hand, histone deacetylases (HDACs) remove acetyl groups from both histone and non-histone proteins, leading to a less accessible chromatin conformation and restraining transcription. HDACis prevent the removal of acetyl groups and maintain accessibility, resulting in increased transcriptional activity 13, 14. This transcriptional accessibility increases expression of anti-cancer genes. Besides, high-resolution mass spectrometry studies identified 3,600 lysine acetylation sites for over 1,700 non-histone targets, which are preferential components of large macromolecular complexes, such as chromatin remodeling, cell cycle, and splicing 15, 16. Thus, epigenetic changes such as post-translational modification by HDACis can lead to having a dramatic effect on the cells' proliferation, differentiation, cell cycle, and cell death. This effect has become one of the reasons why HDACis are used as broad-spectrum anti-cancer agents. Recent studies report that HDACi plays a role in the immune system. For example, entinostat leads to increase of major histocompatibility complex II expression on ovarian cancer cells and differentiate T cells to CD8+ T cells 17. Additionally, entinostat converts immune resistant breast and pancreatic cancer into ICI-responsive tumors by decreasing the number of infiltrating regulatory T cells (Treg) and by suppressing granulocytic-MDSCs (myeloid derived immunosuppressive cells) in the tumor microenvironment (TME) of HER2/neu transgenic breast cancer and metastatic pancreatic cancer mouse models 10, 18. Yet another HDACi, vorinostat has also been known as a stimulator of anti-cancer immune response of B cells through the IFN-γ signal 19. On the other hand, pan-HDAC inhibitors exhibit quite contrasting therapeutic effects through multiple mechanisms: suppressing the destruction of innate immune systems, inhibiting Th1 and Th17 development and expanding Treg 20-22.

CG-745 is a class I and class IIb (HDAC6) inhibitor. Although it has been studied for anti-cancer effects in several cancer models, the role of CG-745 in the immune system is unknown. Therefore, investigation of CG-745's role in the immune system is essential in evaluating the optimal therapeutic potential, thereby, improving treatment schedule for the usage and safety. In this study, we observed CG-745's effects on the immune microenvironment and looked into whether there was induced or prolonged immune activation and altered sub-population of immune cells. Furthermore, CG-745 treatment increased the anti-cancer efficacy of anti-PD-1 antibody in syngeneic mouse models. These findings provide deeper insight into the mechanism of action of CG-745 and serve as an important rationale for the clinical development of this combination.

## Results

### CG-745 increases p21 pression and H3 acetylation

**Figure 1:**
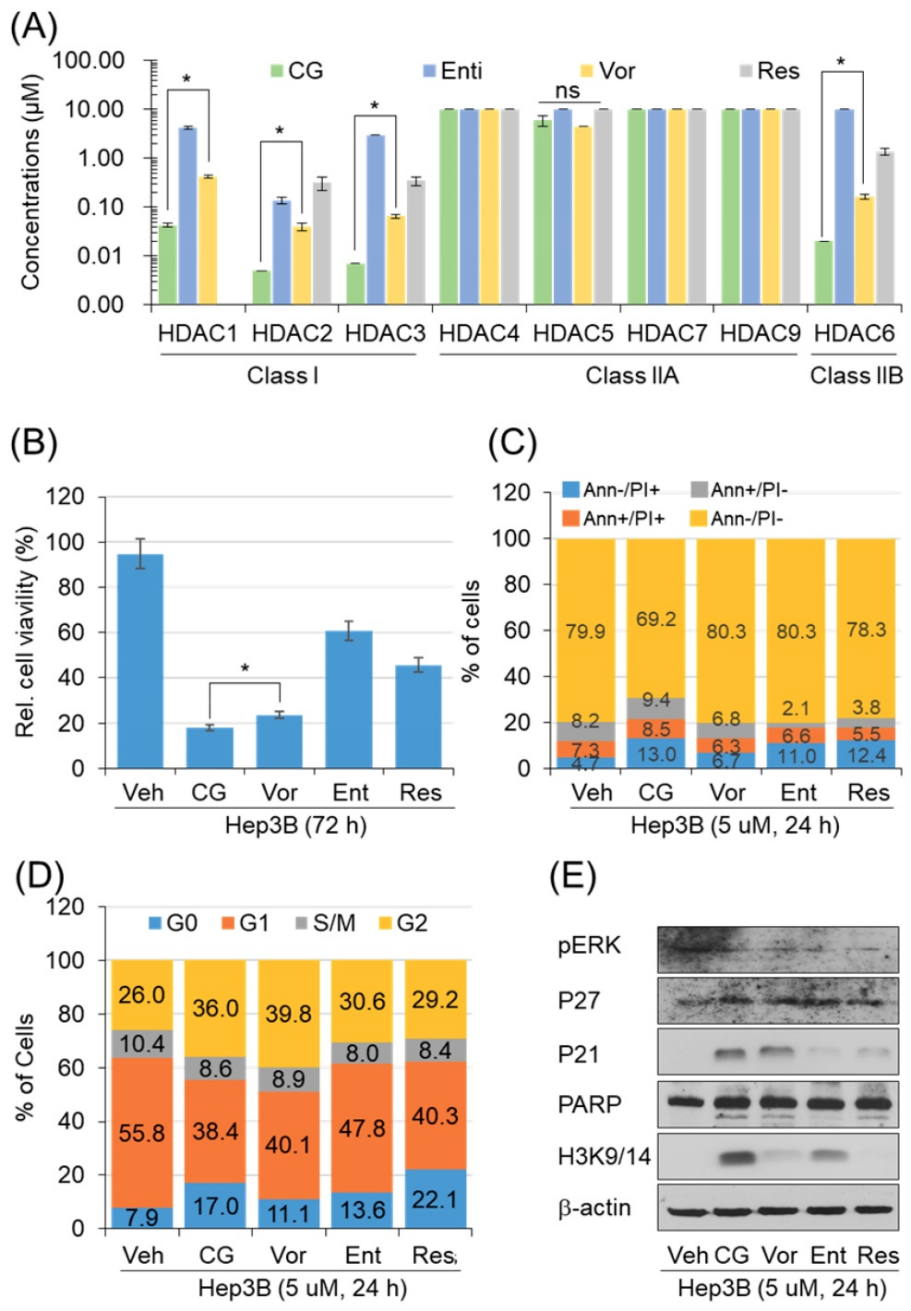
CG-745 shows more potent HDAC inhibition compared to other HDACis and induces cell cycle arrest and cell death relatively early: (A) *In vitro* histone deacetylase inhibitory activity of CG (CG-745), Vor (vorinostat), Ent (entinostat), and Res (resminostat). HDAC activity was analyzed at different HDAC inhibitor concentrations by measuring HDAC substrate fluorescence. Results are shown as means based on experiments performed in triplicate (B) Cytotoxicity of each HDACis on Hep3B cells was analyzed with MTS following 72 hours post-exposure incubation (*p< 0.05); (C, D) Cells were stained by Annexin-V and PI (C), or PI only (D) following 24 hours post-exposure incubation, and samples were analyzed by Attune NxT (Invitrogen) for cell death and cell cycle; (E) Hep3B cells were incubated for 24 hours with HDACis and the total extract was analyzed for expression or phosphorylation of indicated proteins on a text by Western blot.

To address the anti-cancer effect of CG-745 as HDACi, we conducted in vitro enzyme assay. The inhibitory effect against HDACs was compared with those of entinostat, resminostat, and vorinostat. CG-745 showed an inhibitory effect at concentrations below 0.1 μM for HDAC1, 2, 3 and 6 (Figure 1A). The inhibitory concentration (IC50) of CG-745 was about 10 times lower compared to entinostat, vorinostat, and resminostat (Figure 1A). The differences in inhibitory concentrations against HDACs may result in different anti-cancer efficacy. We performed the anti-cancer activity of CG-745 with those of other HDACis in cancer cell line. Hep3B was incubated with each HDACi at the same concentration, and then anti-cancer efficacy was analyzed and represented as Hep3b viability. CG-745 significantly decreased cancer cell viability as compared to those of other HDACis at 72 hours (Figure 1B). As a subsequent study, we compared cell death and cell cycle at an early time (24 h) in order to find out the difference of CG-745 among HDACis. First, we analyzed number of dead cells and found that CG-745 treatment resulted in a greater increase in the number of dead cells under all conditions (Annexin V+/PI-; Annexin V+/PI+; Annexin V-/PI+) compared to those of other HDACis (Figure 1C). We also investigated the differences at the cell cycle level and found that CG-745 treatment arrested cells at G0 and G2 (Figure 1D). Moreover, we were able to confirm meaningful difference on anti-cancer effect not only by p21 expression level but also by PARP cleavage. The increased levels of p21 expression and PARP cleavage by CG-745 were much greater extent compared to those of entinostat, and resminostat. In contrast, vorinostat showed similar results to CG-745, but histone3 lysine 3/14 de-acetylation inhibition was compromised (Figure 1E). Based on these results, it is suggested that the effective anti-cancer activity by CG-745 is due to superior inhibitory effect on HDACs as compared to other HDACis.

### CG-745 affects T cell activation and macrophage polarization

**Figure 2:**
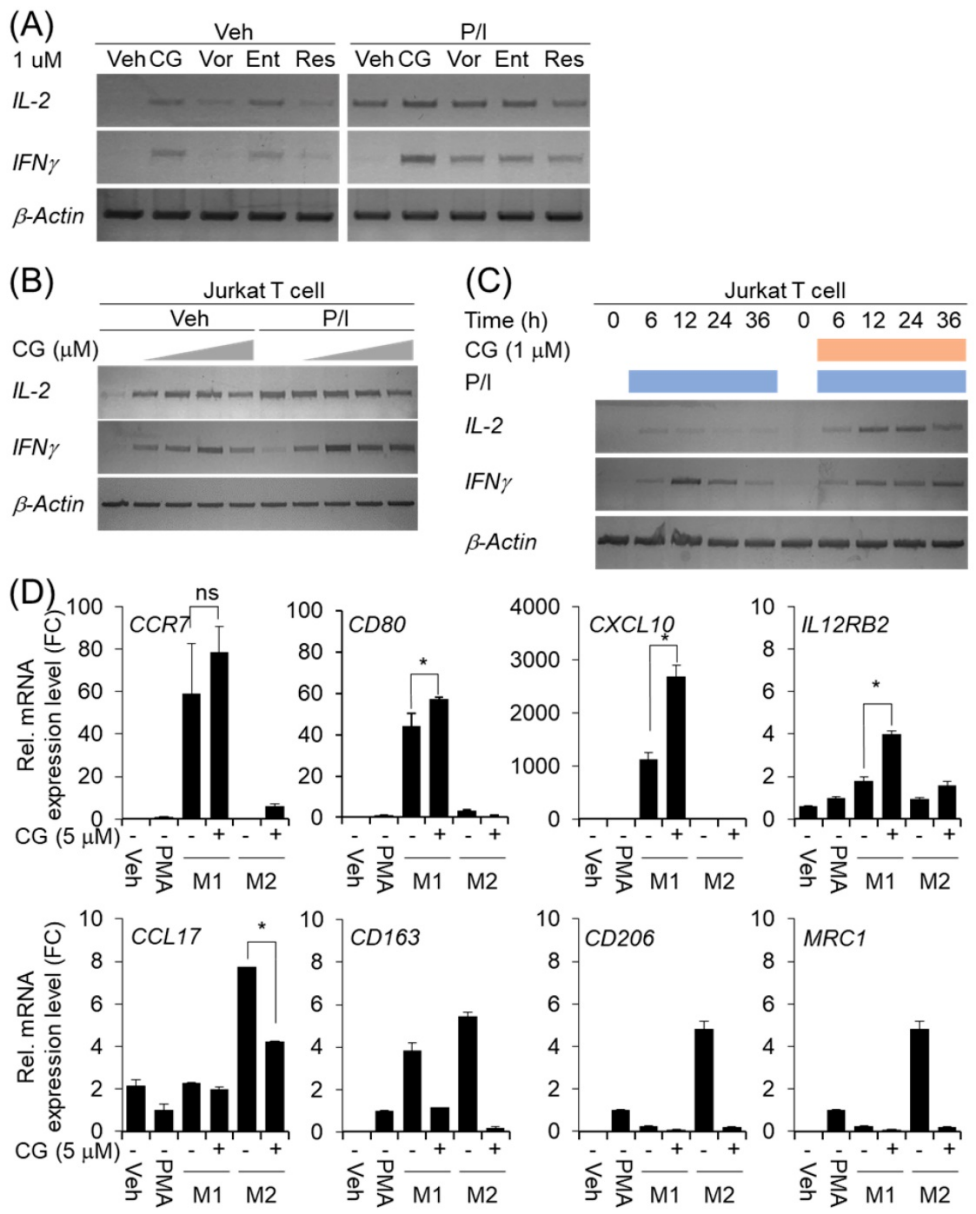
CG-745 induces T cell activation and M2 macrophage polarization suppression: (A) Jurkat T cells were cultured with 1 μM of HDACis for 12 hours and harvested for total RNA extraction followed by PCR; (B) Jurkat T cells were cultured with CG-745 in a dose-dependent manner (0, 0.5, 1, 2.5, 5 μM) for 12 hours and harvested for total RNA extraction followed by PCR; (C) Jurkat T cells were stimulated with PMA (50 nM)/Ionomycin (500 nM) or PMA (50 nM)/Ionomycin (500 nM)/CG-745 (1 μM) and harvested for total RNA extraction in a time-dependent manner; (D) THP-1 macrophages were pre-treated with CG-745 (5 μM) for 1 h and polarized during 48 h with either LPS (10 pg/ml)/ IFN-γ (20 ng/ml) or IL-4 (20 ng/ml)/ IL-13 (20 ng/ml). The mRNA level of macrophage markers (M1 markers: *CCR7, CD80, CXCL10*, and *IL12RB2*; M2 markers: *CCL17, CD163, CD206,* and *MRC1*) was measured by real-time RT-PCR and normalized by *RPL13A*. ns=not significant, *p<0.05.

Many members of the HDAC family are important for T cell development and generation of various aspects in the life of T cells including activation, differentiation, and proliferation 23. The depletion of bystander lymphocytes and regulatory T cells while allowing expansion of antigen-specific secondary response were enhanced by HDACi treatment 21. In the cancer model, entinostat neutralizes MDSC and Tregs 18 and HDAC1 conditional KO T cells increases IFN-γ production 24. In order to find out whether CG-745 affects T cell immunity, we tested the effect of CG-745 on T cell activation and compared with those of vorinostat, entinostat, and resminostat. Jurkat T cell was incubated with CG-745 and other HDACis for 12 hours. Expression of IL-2 and IFN-γ were induced by CG-745 without stimulator, however, vorinostat, and resminostat did not significantly increased IL-2 and IFN-γ expression (Figure 2A). Expression of IL-2 and IFN-γ were further analyzed with PMA (50 nM)/Ionomycin (500 nM) stimulation and each HDACi combination increased IL-2 expression level compared to P/I only (Figure 2A). Interestingly, the expression level of IFN-γ was higher for P/I and CG-745 combination than for other HDACis (Figure 2A). We tested CG-745 with or without P/I to find dosage and time dependency in T cell activation. In the dose-dependent test, IL-2 and IFN-γ expressions were highest at 1 μM and 2.5 μM, respectively without P/I condition. However, additional increase of IL-2 expression was not noticed when co-treated with CG-745 and P/I. In contrast, the expression of IFN-γ was highest at 1 μM of CG-745 with the co-treatment of P/I (Figure 2B). Next, we compared IL-2 and IFN-γ expressions in a time-dependent manner between P/I only or combination of CG-745 and P/I. As a result, CG-745 appeared to cause slower turnover rate while faster expression of IL-2 and IFN-γ with CG-745 and P/I combination condition (Figure 2C).

The tumor microenvironment protects cancer cells from the attack by immune cell by using complicated immune suppression mechanisms. Tumor-associated macrophages (TAMs) are one of the main populations that favor tumor cell growth and survival, thus displaying an M2-like phenotype. On the other hand, M1 macrophages exert antitumor functions. Tumor-derived factors such as VEGF-A and CSF-1 recruit the macrophages in the tumor microenvironment and alter their phenotype in M1 to M2 by secretion of several cytokines including IL-4, IL-13, and VEGF-A 22. We also set out to test for macrophage polarization with CG-745 *in vitro*. When the cells were differentiated into M1 or M2 macrophage from THP1 monocytes with CG-745, expression of CCR7, CD80, CXCL10, and IL12RB2 were higher compared to the control; however, the expression of CCL17, CD163, CD206, and MRC1 was lower compared to the control, indicating that CG-745 enhances M1 polarization but inhibits M2 polarization (Figure 2D). These results suggest that CG-745 promotes immune activation and provides an environment favorable for anti-cancer immunity by modulating M1/M2 polarization.

### CG-745 induces immune microenvironment changes

**Figure 3:**
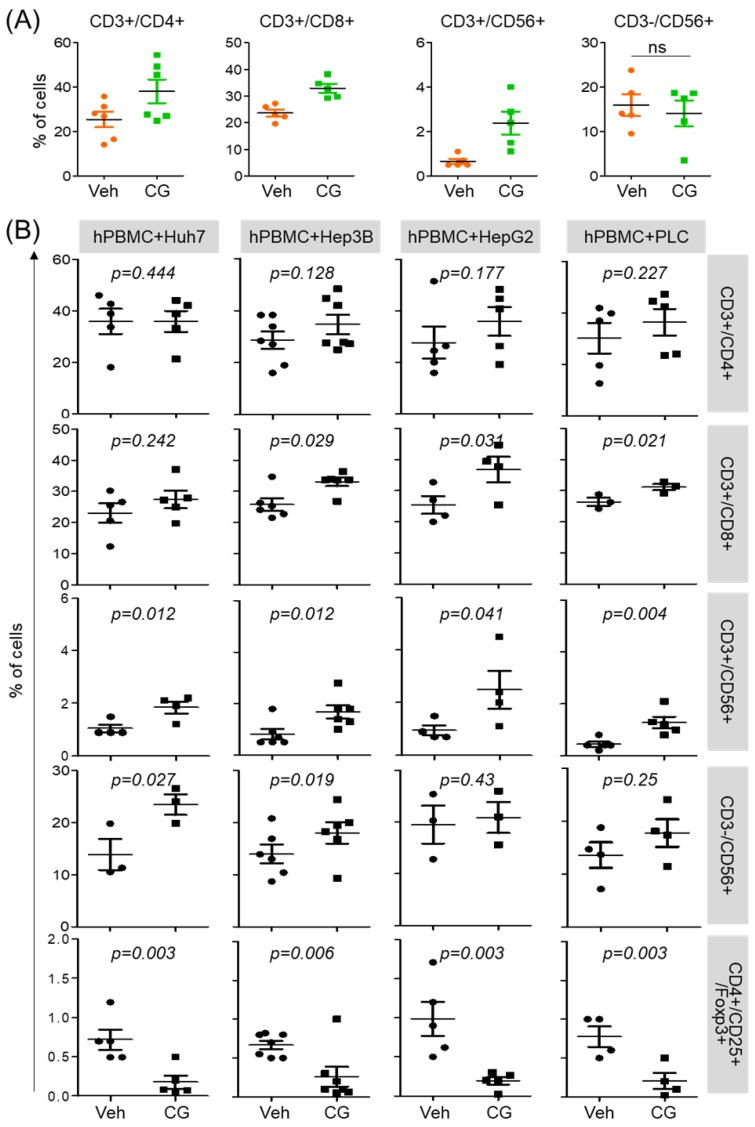
CG-745 increases helper T cells, cytotoxic T cells and natural killer T cells, and decreases Treg: (A) hPBMCs were incubated with CG (CG-745) for 36 hours and a subset of hPBMCs was analyzed using the antibodies indicated in the text; (B) hPBMCs were co-cultured with Huh7, Hep3B, HepG2 or PLC/PRF/5 cells for 36 hours with or without CG, and a subset of hPBMCs was analyzed by Attune Nxt (Invitrogen, USA).

CG-745 influences not only the activation of T cell but also promotes M1 macrophage polarization. To see whether this phenomenon was restricted to T cell activation and macrophage polarization only, we tested immune cell differentiation. hPBMCs was incubated with CG-745 for 36 hours and then analyzed for the sub-population changes of hPBMCs. Helper T cells (CD3+/CD4+), cytotoxic T cells (CD3+/CD8+), and natural killer T cells (CD3+/CD56+) were increased by CG-745, however, the number of natural killer cells (CD3-/CD56+) were not significantly changed (Figure 3A). Sub-population changes of hPBMCs by CG-745 were further analyzed in a co-culture system with human cancer cell lines. Each of co-culture system showed the increase of CD3+/CD8+, CD3+/CD56+ and CD3-/CD56+ cells, and the decrease of Treg (CD4+/CD25+/Foxp3+) (Figure 3B). The effect of CG-745 on immune cell subpopulations changes was broader when compared to single culture conditions. These results demonstrate that CG-745 induces immune cell subpopulation changes that can effectively increase anti-cancer effect when hPBMCs are present with cancer cells.

### CG-745 induced subpopulation changes of immune cells promote hPBMCs killing-activity

**Figure 4:**
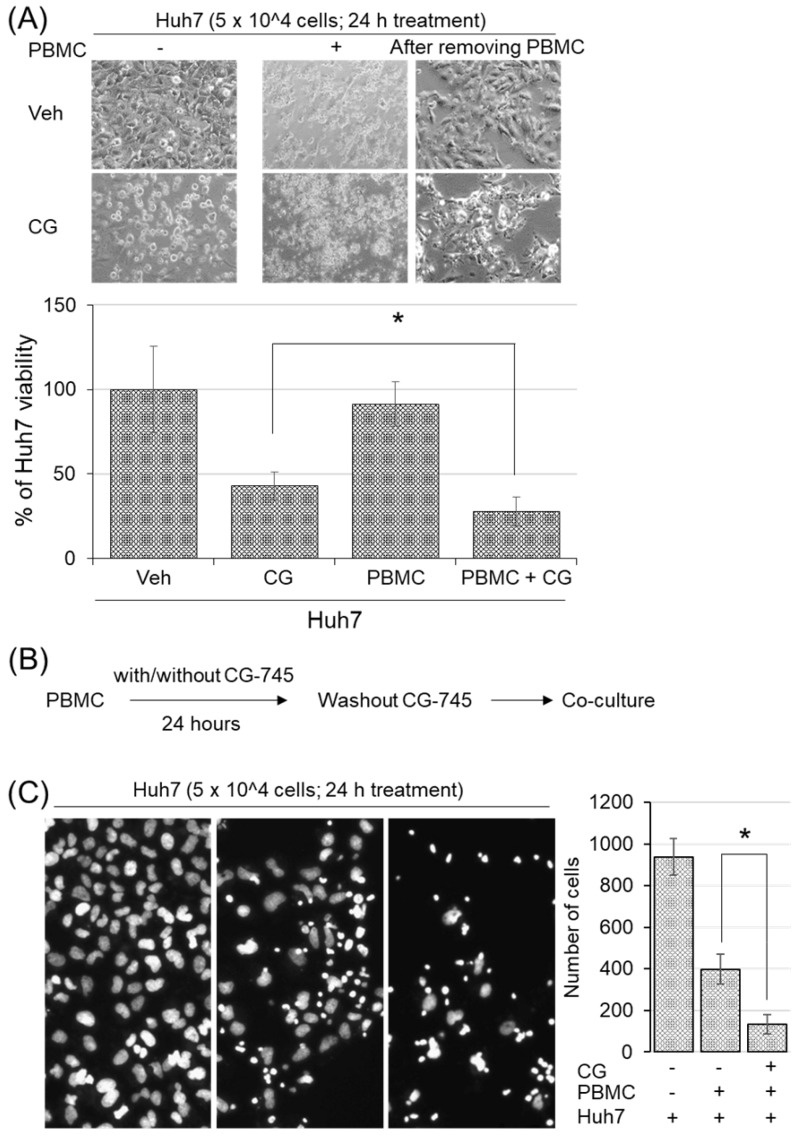
CG-745 increased the killing activity of hPBMCs against Huh7: (A) Huh7 cells were co-cultured for 24 hours with or without hPBMCs in the presence or absence of CG (CG-745) (5 μM). Images were obtained by fluorescence microscopy and the remaining number of cells were counted by Image J software; (B) Schematic diagram of the killing activity assay; (C) hPBMCs with/without CG (5 μM) were pre-incubated for 24 hours, and co-cultured with Huh7 cells for another 24 hours, then analyzed the number of remaining Huh7 cells. The images were obtained by fluorescence microscopy (Leica) and analyzed by Image J software. * *p* < 0.05

To examine whether CG-745 induced immune microenvironment changes affect anti-cancer immunity, we conducted cytotoxicity assay in the co-culture system. Huh7 cells were incubated with hPBMCs and CG-745 (5 μM), and then measured the viability of Huh7 cells after 24 hours incubation. The killing effect of hPBMCs on Huh7 cells was not observed without CG-745 condition, being only 27.88% of Huh7 remained alive when hPBMCs were co-treated with CG-745 (Figure 4A). The viability of Huh7 was 15% lower for the co-treatment of CG-745 and hPBMC, compared with that for the CG-745 treatment (42.93%) only. In a further study, to eliminate direct effect of CG-745 against Huh7 cells viability, we pre-incubated the hPBMCs with CG-745 for 24 hours and then co-cultured with Huh7 cells for another 24 hours (Figure 4B). As we expected, the number of live Huh7 cells was less in pre-incubated hPBMCs with CG-745 than in pre-incubated hPBMCs with vehicle (Figure 4C). All these results suggest that CG-745 can synergistically increase anti-cancer effect via direct killing of cancer cells and inducing immune microenvironment changes.

### Antitumor efficacy of anti-PD-1 antibody is increased by CG-745 in syngeneic tumor mouse models

**Figure 5:**
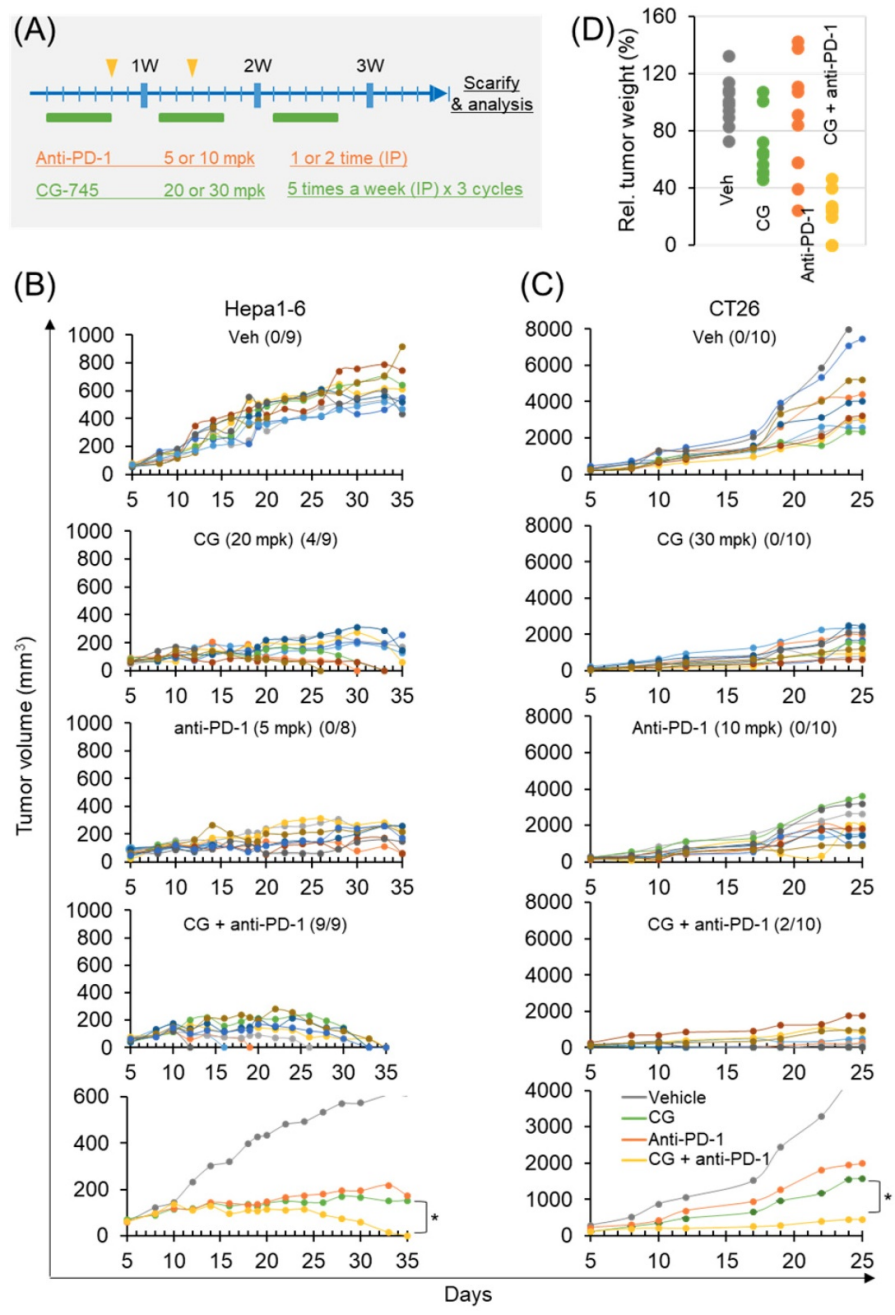
Anti-cancer efficacy of PD-1 antibody was increased by CG-745 co-treatment in Hepa1-6 and CT26 syngeneic mouse models: (A) A schematic diagram of the treatment schedule; (B, C) Hepa1-6 (B) or CT26 (C) syngeneic mice were injected with 20 (B) or 30 mg/kg (C) of CG (5 days per week for 3 weeks intraperitoneally (IP)). The anti-PD-1 antibody was treated alone or in combination with CG as indicated by the arrow and rectangle in Figure 5A. Tumor volume at indicated time points after treatment was plotted. The line graph shows tumor volume of individual mouse or average of mice (bottom); The numbers in parentheses indicate tumor free mice/total mice. (D) Representative tumor weights at the end of the experiment. Tumor tissues were collected from individual mouse.

To confirm the role of CG-745 on tumor growth *in vivo* when combined with an ICI, we used two syngeneic mouse models. The dosages in each syngeneic mouse model were designed at different concentrations based on previous tumor growth tests (Figure 5A). In the subcutaneous Hepa1-6 mouse model, CG-745 alone produced complete tumor clearance response in 4 mice at days 25, 30, and 33, and no tumors were observed in these mice until sacrifice on day 50 (Figure 5B). Anti-PD-1 treated mice did not show complete tumor clearance response. However, the final results of tumor growth inhibition were similar to those of CG-745 treated mice (Figure 5B). As we expected, the anti-cancer efficacy of anti-PD-1 with CG-745 combination showed tumor clearance response in all mice on day 35 (Figure 5B, bottom). In the case of CT26 syngeneic mouse model, it showed very rapid tumor growth and CG-745 or anti-PD-1 mono-treatment did not show tumor clearance response. The CT26 syngeneic mouse model also showed effective tumor growth suppression including complete tumor clearance (two mice) when treated with CG-745 and anti-PD-1 combination (Figure 5C). In a further analysis, the comparison for tumor weights revealed that while tumor weights of each individual mouse treated with CG-745 were very similar, the tumor weights of the mouse in the anti-PD-1 treated group varied widely. These results suggest that effect on anti-cancer immunity by CG-745 reduces the difference of individual immune responses, thereby helping anti-PD-1 anti-cancer activity.

### CG-745 increases infiltrated lymphocyte and decreases MDSCs and M2 macrophage in vivo syngeneic tumor mouse model

**Figure 6:**
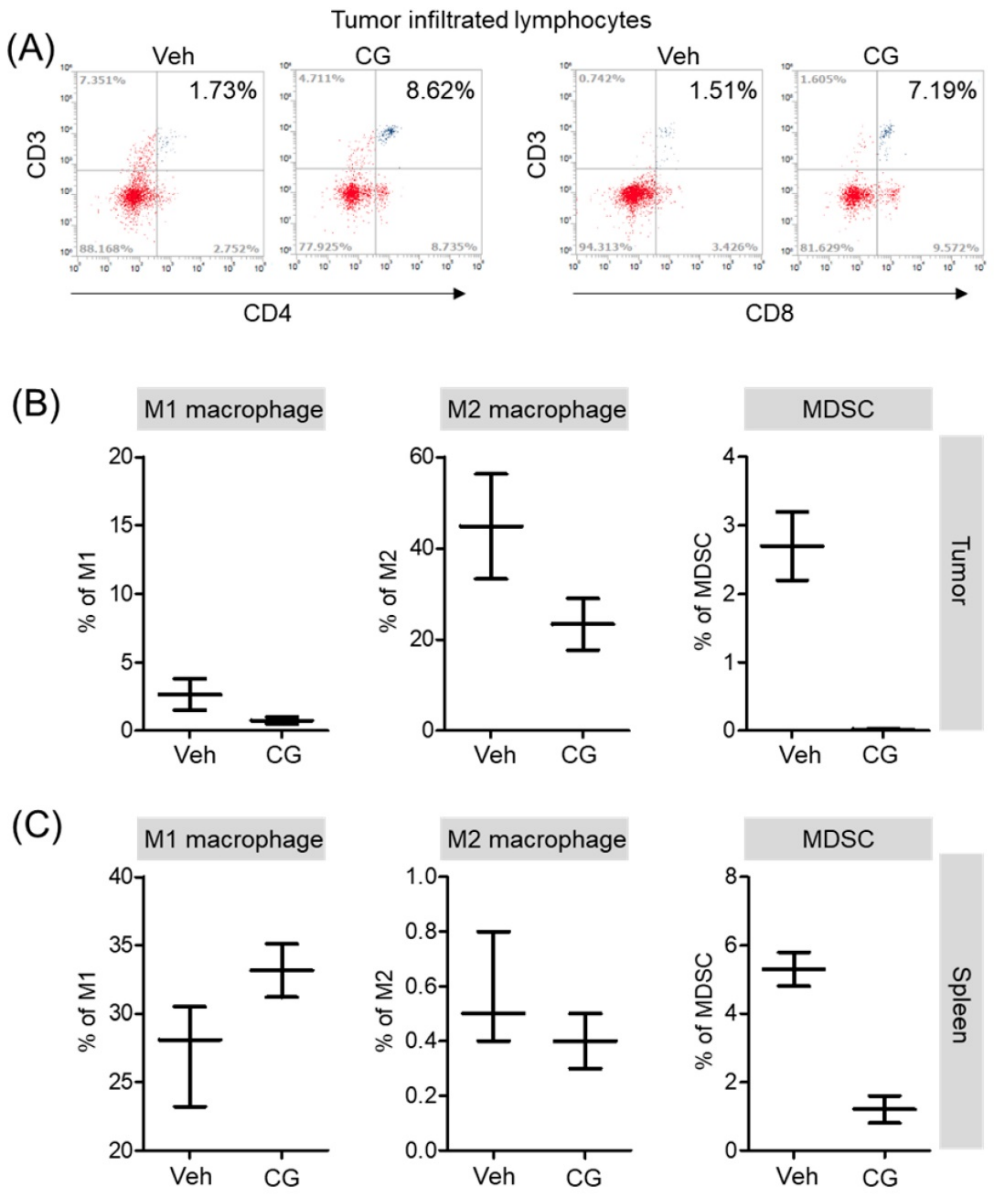
The proportion of MDSCs and M1/M2 macrophage were changed by CG-745 treatment in Hepa1-6 syngeneic mouse model: (A-C) Hepa1-6 inoculated C57BL/6 mice were treated with vehicle or 15 mg/kg/day of CG (CG-745) by intraperitoneally for 5 days/week for 3 weeks. Tumor (A, B) and spleen (C) were harvested from each individual mouse. Cells were stained with fluorescence-conjugated antibodies specific to CD3, CD4, CD8, CD25 and Foxp3, and subjected to flow cytometry analysis. The graph shows means with error bars. Error bars represent standard deviations for 3 samples in each group. Results are representative of three separate experiments (A). *p* < 0.05.

Both *in vitro* and *in vivo*, CG-745 showed a positive role in the immune system against cancer. Additionally, we confirmed that CG-745 increases anti-cancer effects of anti-PD-1 in two syngeneic mouse tumor models. The immune microenvironment of tumor tissue is one of the hurdles for anti-cancer therapy of ICIs. Therefore, the enhancement of the anti-cancer efficacy of anti-PD-1 is likely to be caused by CG-745 through the induction of the immune environment changes. To examine immune microenvironment changes of mouse by CG-745, mice were selected randomly and the tumor tissues were isolated. Consistently *in vitro*, T cells in the tumor microenvironment were higher in CG-745 (CD4 (8.62%) and CD8 (7.19%)) than in vehicle group (CD4 (1.73%) and CD8 (1.51%)) (Figure 6A). The analysis macrophages and myeloid-derived suppressor cells (MDSCs) in tumor (Figure 6B) and spleen (Figure 6C) showed that the numbers of MDSCs were drastically decreased by CG-745 in both, tumor and spleen. The M2 macrophages (27.9%) in the CG-745 treated group were lower than in the vehicle treated group (44.8%). The M1 macrophage in CG-745 was also slightly lower than that of the vehicle treated group. Conversely, in the spleen, the number of M1 macrophages were higher in the CG-745 treated group (33.1%) than in the vehicle treated group (27.9%). The number of M2 macrophages in the spleen was low in CG-745 group. All these results suggest that the changes induced by CG-745 in the immune microenvironment are beneficial to immune oncology agents such as anti-PD-1 antibodies.

## Discussion

Histone deacetylase inhibitors (HDACis) are potent epigenetic modulators that have various therapeutic potential and have pleiotropic effects at the cellular and systemic levels 25. While HDACis in anti-cancer therapy are commonly known as apoptosis inducers through anti-cancer gene expression and cell cycle arrest, recent studies have shown anti-tumor efficacy of HDACis in certain cancer types 26-29. CG-745 is a HDAC inhibitor, and it has been reported as a potent anti-cancer agent in cholangiocarcinoma, pancreatic cancer, prostate cancer, and non-small cell lung cancer 30-33. In cholangiocarcinoma, CG-745 is known to target the Hippo pathway via upregulation of miR-509-3p expression 33. Also, reduction of the ATP-binding cassette-transporter genes, especially multi-drug resistance protein 3 and 4 which also resist CG-745, induce pancreatic cancer cells to sensitively respond to gemcitabine. Consequently, CG-745 leads to a synergistic anti-tumor effect on pancreatic cancer cells when combined with gemcitabine/erlotinib in mouse tumor model 32.

Here, we have found another role of CG-745 in anti-cancer function. CG-745 induces and prolongs the T cell activation, while increasing the population of CD3+/CD8+ T cells, CD3+/CD56+, and CD3-/CD56+ cells and decreasing the populations of CD4+/CD25+/Foxp3+, and MDSC *in vivo* and *in vitro*. Furthermore, CG-745 enhances M1 polarization but inhibits M2 polarization *in vitro*.

The changes in the composition of immune cells by CG-745 are not the only cause of anticancer effects. Induction of immune activity by CG-745 was confirmed by increasing cytotoxicity of PBMC and increasing* IFNγ* expression of Jurkat T cells. Histone3K9ac/K14ac induces the transcriptional activity of *IFNγ* gene, as well as the activity of genes associated with T cell function and development 34. Acetylation of histone H3 was also an important factor during the development of naïve CD8 + T cells into memory T cells 35. Therefore, it can be thought that the change of immune cell composition is due to activation of the immune cell by CG7-45 induced landscape change of histone acetylation.

Due to the fact of individual HDACs which are targeted by HDACis have distinct roles in immune responses, not all HDACis are positively associated with anti-cancer immunity. In animal models, HDACis are therapeutic for several inflammatory diseases 36 and compromise host defense 37-39. Even, loss of HDAC function has also been linked to chronic lung disease in human 40. These contradictory effects might reflect the distinct roles of individual HDACs in the immune responses. Therefore, the novel discovery of CG-745 in anti-cancer immunity suggests an excellent combination partner of various cancer immunotherapy and supports the reason why CG-745 shows the excellent synergistic efficacy in the combination treatment of various mouse tumor models such as in cholangiocarcinoma, pancreatic cancer, prostate cancer, and non-small cell lung cancer 30-33.

From combination studies, CG-745 has shown to enhance the immune system's anti-cancer effects and increase anti-cancer efficacy of anti-PD-1 antibody *in vivo*. Cells in the body continue to make spontaneous mutations, some of which target proto-oncogenes or tumor suppressor genes. These are blocked by an immune system that can identify cancer itself and eliminate possibilities of further progression 41. Thus, a patient who already has cancer means that the anti-cancer immunity is incomplete or it has cancer that has the potency to avoid anti-cancer immunity. The cancer dominance over the immune system is a major obstacle in designing effective anti-cancer therapeutic strategies. In order to counter-attack, the new concept of immune checkpoint inhibitor (ICI) had been introduced and succeeded in cancer treatment. However, if one of the reasons of cancer development in a patient is an abnormality of the immune system, then the ICI therapy will not have a full effect. In addition, since the TME contains several immune suppressor cells such as Tregs, MDSCs, and tumor-associated macrophages (TAM) 42, 43, there is a limit to the induction and persistence of immune activation by ICI. In fact, while ICI has been considered to have potentially high anti-cancer effects in melanoma, the actual response rate of ICI was less than 30%, which was similar to that of conventional chemotherapy in most solid tumors. Additionally, although ICIs had achieved clinical responses , only a minority of patients exhibited durable responses 1, 44.

As a result of the mixed PBMC response analysis using several types of cancer cells, it was found that there was a difference in immune cell composition between type of cancer cells when CG-745 was treated. In addition, in the tumor growth inhibition test using a mouse, it can be seen that the change of immune cell composition varies slightly between individuals and carcinomas. However, the composition of immune suppressor cells was consistently decreased by CG-745 treatment. The results of this study support the notion that core histone deacetylation constitutes more functions than a simple trigger for anti-cancer gene induction. We report here for the first time that CG-745 influences TME changes, inducing immune activation and enhancement of immune cancer activity. We, therefore, believe that CG-745 as an anti-cancer agent has the potential to be an excellent combination partner for immunotherapy to overcome the limitation of ICI treatment.

## Materials and Methods

### Reagents and antibodies

The following antibodies were used in this study: anti-p21 (2946S; Cell Signaling Technology, Danvers, MA, USA), anti-PARP (9532S; Cell Signaling Technology), anti-Acetyl-Histone H3 (Lys9/Lys14) (9677; Cell Signaling Technology), anti-β-actin (SC47778; Santa Cruz, CA, USA), anti-pERK (4370S; Cell Signaling Technology), anti-p27 (3686S; Cell Signaling Technology), anti-mouse PD-1 (BP0146; BioXcell, West Lebanon, NH, USA), anti-human CD3 (35-0036-42; Invitrogen, Waltham, MA, USA), anti-mouse CD3(11-0037-42; Invitrogen), anti-human CD4 (12-0049-42; Invitrogen), anti-mouse CD4 (12-0041-82; Invitrogen), anti-human CD8 (MA1-19626; Invitrogen), anti-mouse CD8 (12-0081-82; Invitrogen), anti-mouse CD11b (12-0112-82; Invitrogen), anti-human CD25 (11-0257-42; Invitrogen), anti-mouse CD25 (MA5-17815; Invitrogen), anti-human CD56 (11-0566-42; Invitrogen), anti-mouse CD86 (15-0862-81; eBioscience, San Diego, CA, USA), anti-mouse CD206 (141704; BioLegend, San Diego, CA, USA), anti-mouse Gr-1 (11-5931-82; eBioscience), anti-human Foxp3 (35-4776-42; eBioscience), anti-mouse Foxp3 (35-5773-82; eBioscience). The following secondary antibodies were used: HRP conjugated anti-rabbit IgG (7074P2; Cell Signaling Technology), HRP conjugated anti-mouse IgG (7076; Cell Signaling Technology), and HRP conjugated anti-goat IgG (sc-2354; Santa Cruz). The following HDAC inhibitors were used: vorinostat (V-8477; LC Laboratories, MA, USA), entinostat (E-3866; LC Laboratories, MA, USA), resminostat (S2693; Selleckchem, TX, USA). CFSE (C34554; Waltham, MA, USA), and Hoechst 33342 (H3570; Invitrogen) was used for image analysis and cell counting. C57BL/6 and BALB/c mice (5~6 weeks old) were acquired from DAHAN Bio for use in the tumor growth inhibition study. Additionally, 5 x HOT FIREPol Blend Master Mix (042-25-00120; SolisBiodyne, Tampere, Finland), SuperScript VILO Master Mix (11755-050; Invitrogen) were acquired for PCR use.

### Cell culture

MEM (11095-080; Gibco, Grand Island, NY, USA) for Hep3B (CRL-1634; ATCC, Manassas, VA, USA), DMEM (11965-092; Gibco, UK) for Huh7 (CRL-1634; ATCC), and RPMI1640 (22400-089; Gibco, NY, USA) for THP-1 cells (CRL-1634; ATCC,) and Jurkat T (CRL-1634; ATCC) were used for cultivation of each cell line. Each medium was supplemented with 10% FBS (16000-044; Gibco) and penicillin/streptomycin (100 μg/ml; Invitrogen). THP-1 cells were cultured in RPMI1640 containing 10% FBS and supplemented with 0.05 mM ß-mercaptoethanol (M3148, Sigma, MO, USA). Cells were cultured under a humidified 5 % (v/v) CO2, atmosphere at 37 °C.

### Macrophage polarization assay

Human monocytic THP-1 cells were cultured in Roswell Park Memorial Institute medium (RPMI 1640) (22400-089; Gibco, NY, USA) supplemented with 10 % fetal bovine serum and 50 pM β-mercaptoethanol (M3148; Sigma-Aldrich, MO, USA). THP-1 monocytes were treated with 100 nM phorbol 12-myristate 13-acetate (PMA) (P8139; Sigma-Aldrich, MO, USA) to differentiate into macrophages for 24 h, followed by 24 h incubation with PMA-free RPMI medium. Macrophages were untreated or pretreated with CG-745 (5 μM) for 1 h. M1 macrophages were obtained by 48 h incubation with 20 ng/ml IFN-γ (285-IF; R&D system, MN, USA) and 10 pg/ml LPS (L2880; Sigma-Aldrich, MO, USA). While M2 macrophages were polarized by 48 h incubation with 20 ng/ml IL-4 (204-IL; R&D system, MN, USA) and 20 ng/ml IL-13 (213-ILB; R&D system, MN, USA). After incubation macrophages were harvested for real-time qPCR.

### Real-time and Semi-quantitative PCR

Total RNA was extracted using Trizol reagent (15596018; Invitrogen) according to the manufacturer's instructions. For real-time qPCR, cDNA was synthesized using SuperScript IV First-Strand Synthesis System, following the manufacturer's instructions. Real-time qPCR was performed in triplicate using SsoAdvanced™ Universal SYBR® Green Supermix (BR172-5272; Bio-Rad, CA, USA,) on a CFX Connect™ Real-Time PCR Detection System (Bio-Rad, CA, USA, CFX Connect™ Optics module). Relative expression was normalized using ribosomal protein L13a (RPL13A) or β-actin. Semi-quantitative PCR was carried out using HOT FIREPol Blend Master Mix according to the manufacturer's instructions. The primer sequences used were as follows: CCR7 forward, 5'-AGC GTC ATG GAC CTG GGT ATG C-3', and reverse, 5'-CAA CAC GAC CAG CCC ATT GC-3'; CD80 forward, 5'-CCC AGG AAC ACC CTC CAA TC-3', and reverse, 5'-ACG TGG ATA ACA CCT GAA CAG A-3'; CXCL10 forward, 5'-GTC CAC GTG TTG AGA TCA TTG CT-3', and reverse, 5'-GCC TCT GTG TGG TCC ATC CT-3'; IL12RB2 forward, 5'-AAA GGA CAT CTG CGA GGA AAG TTC-3', and reverse, 5'-CGA GGT GAG GTG CGT TTA TGC-3'; CCL17 forward, 5'-TGC CAT CGT TTT TGT AAC TGT GC-3', and reverse, 5'-CTG GGG TGA GGA GGC TTC AA-3'; CD163 forward, 5'-CAG CGG CTT GCA GTT TCC TC-3', and reverse, 5'-TGG CCT CCT TTT CCA TTC CAG A-3'; CD206 forward, 5'-GAC ACG ATC CGA CCC TTC CT-3', and reverse, 5'-TGT TCA GGG CGA TCC ACA CA-3'; mannose receptor C type 1 (MRC1) forward, 5'-ACC TCA CAA GTA TCC ACA CCA TC-3', and reverse, 5'-CTT TCA TCA CCA CAC AAT CCT C-3'; RPL13A forward, 5'-GAA GGT GGT GGT CGT ACG CT-3', and reverse, 5'-CGC CCC AGA TAG GCA AAC TTT C-3'; IL2 forward, 5'-ATG CTG ATG AGA CAG CAA CCA-3', reverse, 5'-GAG CCC CTA GGG CTT ACA AAA-3', IFNγ forward, 5'-CTG TTA CTG CCA GGA CCC AT-3', reverse, 5'-TGC TTT GCG TTG GAC ATT CA-3'. The thermal cycling conditions were as follows: pre-denaturation at 95 °C for 30 sec, denaturation at 95 °C for 15 sec, annealing/extension at 62 °C for 30 sec.

### Cell cycle analysis

Hep3B cells were harvested and washed in PBS. To fix the cells, cold 70% ethanol was added as drop wise to the pellet while vortexing. After standing 30 minutes at 4 °C, the cells were washed 2 x in PBS. Then cells were treated with ribonuclease (5 μg) and PI (propidium iodide) (20 μg). The cell cycle was analyzed by using FACS AttuneNxT (Invitrogen by Thermo Fisher Scientific).

### Analysis of apoptosis by flow cytometry

Hep3B cells were seeded into 96-well plates at a density of 5×10^3^ per well (100 μl) and treated with the following: negative control (DMSO), CG (CG-745), Enti (Entinostat), Vori (Vorinostat), and Res (Resminostat) 5 μM. The cells were treated for 72 h. For the cytotoxicity assay, the CellTiter 96 Aqueous One Solution Cell Proliferation Assay kit (Promega Inc, #G3581, Madison, USA) was used following the manufacturer's instruction. Briefly, 20 μl of the MTS [3-(4,5-dimethylthiazol-2-yl)-5-(3-carboxymethoxyphenyl)-2-(4-sulfophenyl)-2H-tetrazolium] reagent was added into each well and cells were incubated at 37°C for 2 h. The absorbance was detected at 490 nm with a microplate reader SpectraMax i3X (Molecular Devices, Sunnyvale, CA, USA). Each biological experiment has three technical replicates, and the cell viability was normalized compared with that of the DMSO treated (negative control, NT) cells.

### Human PBMC isolation

Human Peripheral Blood Mononuclear Cells (PBMCs) were obtained from the Korean Red Cross, based on the protocol approved by their Institutional Review Board (IRB). The protocol to freshly isolate PBMCs from healthy donors is based on using Ficoll-Paque Plus (GE Healthcare, Chicago, IL, USA) according to manufacturer's protocol. Briefly, freshly collected blood was diluted with two-fold PBS (Ca/Mg, -/-). The diluted blood was then gently loaded on top of 20 mL Ficoll-Pague Plus in a 50 mL tube. According to the manufacturer's protocol, the tube was spun at 2000 rpm for 20 minutes at room temperature. The supernatant was removed and the cells collected with another spin at low speed, 1200 rpm for 5 minutes. Once collected, the cell pellet was subjected to red blood cells lysis by applying Red Blood Cell Lysis Buffer (Biolegend) for 5 minutes. The remaining cells were then washed 2-3 times with PBS. Finally, the PBMCs were collected and resuspended in RPMI 1640 with 10% FBS and Pen/Strep. Once PBMCs were isolated, they were either cultured briefly in the above medium or were added to target cells immediately.

### Animal experiments

Female C57BL/6 (6 weeks of age) mice were purchased from Charles River (Seoul, Korea) and maintained under specific pathogen-free conditions at 23°C. They were quarantined for the first 7 days after arrival. We assigned mice randomly to either the experimental or control group. The mice had access to food and water ad libitum. All experiments with mice were approved by the Animal Care Committee of CrystalGenomics. The mice were subcutaneously inoculated with 1×10^6^ cells/mouse Hepa1-6 cells on the left flank. The cells were suspended at 1:1 (vol/vol) ratio with Matrigel (BD Biosciences, cat. no. 356234). Seven days after tumor cell inoculation, mice had been randomly divided into groups. Tumor diameter was measured every 2-3 days, and tumor volume (in mm^3^) was calculated using a caliper. Tumor size was expressed as tumor volume using the formula: tumor volume (mm^3^) = (major axis) x (minor axis)^2^ x 0.5. Animals were killed when their tumors were more than 20 mm in the longest dimension. They were considered tumor-free when tumor dimensions were less than 1 mm and kept under observation for at least 50 days. Hepa 1-6 cells were purchased from the American Type Culture Collection (ATCC, CRL-1830, Manassas, VA, USA). The cells were cultured in H-DMEM medium supplemented with 10% FBS. For animal experiments, cells were passaged two to five times. Cells in log phase were washed and resuspended in PBS immediately before injecting them into mice.

### Isolation of cells from tumor tissue

Tumor-infiltrating lymphocytes (TILs) were isolated from tumors as described previously with minor modifications. Briefly, tumors were cut into fragments 2-3 mm in width, and the fragments were incubated in RPMI 1640 medium-containing 10% FBS, collagenase type I (300 U/ml; Gibco, Carlsbad, CA) and DNase I (50 U/ml; Sigma, St Louis, MO) at 37°C for 90 min. Thereafter, the digested fragments were teased through steel mesh and washed in PBS.

### FACS analysis

For fluorescence-activated cell sorter (FACS) analysis, immune cells were blocked with Fc receptor-blocking antibody 2.4G2 for 20 min at 4 °C and stained with antibodies indicated in text for another 20 min. After washing once with PBS, samples were analyzed with a FACS AttuneNxT (Invitrogen by Thermo Fisher Scientific).

### Western blotting

Samples of approximately 30 μg per extract were electroblotted onto PVDF membranes using a Mini Trans-Blot Cell (Bio-Rad, CA, USA). Each membrane was blocked in 5% skim milk (1 h), rinsed, and incubated overnight at 4 °C with the indicated antibodies in TBS containing 0.05% Tween-20 (TBS-T) and 3% skim milk. The excess primary antibody was removed by washing the membrane with TBS-T four times for 5 min. The membrane was then incubated with 0.1 μg/ml peroxidase-labeled secondary antibody for 2 hours at room temperature. After three washes in TBS-T, the membranes were treated with WesternBright Sirius ECL HRP substrate (K-12043-D10; Advansta, CA, USA) and visualized using a LAS-3000 (Fujifilm, Japan).

### In vitro histone deacetylase inhibition assay

*In vitro* histone deacetylase (HDAC)-inhibitory activity of CG-745, vorinostat, entinostat, and resminostat were analyzed at various concentrations by measuring fluorescence of HDAC substrate. CG-745, vorinostat, entinostat, and resminostat were diluted. Purified HDACis, and fluorescent HDAC substrate was added. The reaction was allowed to proceed for 10 min at 25°C and then the mixture was assayed for HDAC activity. Results are shown as means based on experiments performed in triplicate.

### Mixed PBMC reaction assay

Mixed PBMC reaction assay was performed based on a previous report 45. Dissociated adherent target cells (Huh7, KLM-1, or PK45P) were seeded at a density of 5x10^4^ cells/well after stained with CFSE (ThermoFisher, CellTrace CFSE cell Proliferaion kit, #C34554) according to the manufacturer. After O/N, 100 µL of medium was removed from each well, 5 x 10^5^ of hPBMC/100 µL was added and further cultured for 12 hours. The number of remaining target cells were counted for comparison of killing efficacy by using ImageJ particle analysis plug-in.

### Statistical analysis

All statistical differences, tumor areas, mean normalized gene expression, mean fluorescent intensity (MFI), and percentages of infiltrating immune cells, were determined by non-parametric test (Wilcoxon rank sum test).
